# Hormonal Environment and HER2 Status in Extra-Mammary Paget’s Disease (eMPD): A Systematic Literature Review and Meta-Analysis with Clinical Considerations

**DOI:** 10.3390/diagnostics10121040

**Published:** 2020-12-03

**Authors:** Giuseppe Angelico, Angela Santoro, Frediano Inzani, Patrizia Straccia, Damiano Arciuolo, Antonino Mulè, Michele Valente, Saveria Spadola, Nicoletta D’Alessandris, Giorgia Garganese, Federica Cianfrini, Alessia Piermattei, Giovanni Scambia, Gian Franco Zannoni

**Affiliations:** 1 Unità di Gineco-Patologia e Patologia Mammaria, Dipartimento Scienze della Salute della Donna, del Bambino e di Sanità Pubblica, Fondazione Policlinico Universitario A. Gemelli IRCCS, Largo A. Gemelli 8, 00168 Roma, Italy; giuangel86@hotmail.it (G.A.); angela.santoro@policlinicogemelli.it (A.S.); patrizia.straccia@guest.policlinicogemelli.it (P.S.); damiano.arciuolo@policlinicogemelli.it (D.A.); antonino.mule@policlinicogemelli.it (A.M.); dr.valente.m@gmail.com (M.V.); saveriaspadola@hotmail.it (S.S.); ndalessandris@gmail.com (N.D.); federica.cianfrini@policlinicogemelli.it (F.C.); alessia.piermattei@policlinicogemelli.it (A.P.); gianfranco.zannoni@unicatt.it (G.F.Z.); 2 Unità di Ginecologia Oncologica, Dipartimento Scienze della Salute della Donna, del Bambino e di Sanità Pubblica, Fondazione Policlinico Universitario A. Gemelli IRCCS, Largo A. Gemelli 8, 00168 Roma, Italy; ggarganese@gmail.com (G.G.); giovanni.scambia@policlinicogemelli.it (G.S.); 3 Istituto di Clinica Ostetrica e Ginecologica, Università Cattolica del Sacro Cuore, Largo A. Gemelli 8, 00168 Roma, Italy; 4 Istituto di Anatomia Patologica, Università Cattolica del Sacro Cuore, Largo A. Gemelli 8, 00168 Roma, Italy

**Keywords:** estrogen receptor, progesterone receptor, androgen receptor, HER2, extra-mammary Paget disease, vulvar cancer, immunohistochemistry

## Abstract

Background. Extra-mammary Paget’s disease (EMPD) is a rare neoplasm of epithelial origin, whose precise incidence is not clear. Starting from what is already known, we performed a systematic review and meta-analysis to investigate in male and female patients the immunohistochemical expression of biological markers that could serve as potential prognostic/therapeutic factors, including only human epidermal growth factor receptor 2 (HER2/neu), Estrogen Receptor (ER), Progesterone Receptor (PR), and Androgen Receptor (AR). Methods. A literature search was performed of the PubMed, Scopus, and Web of Science databases for English-language studies published from January 2000 to June 2020. Results. A total of 27 studies with 713 patients assessed the role of HER2/neu, AR, ER, and PR expression in male and female with EMPD. The overall rate of HER2/neu expression was 30%, the expression’s rate for ER and AR was 13% and 40%, respectively, and the overall rate for PR was 8%. The subgroup analysis revealed that there is a different expression of molecular markers between male and female patients. Conclusions. This study revealed that AR status and HER2/neu overexpression/amplification have been shown as two fundamental pathogenetic pathways in both female and male patients affected by EMPD.

## 1. Introduction

Extramammary Paget disease (EMPD) was first described by Crocker in 1889 in a man affected from bladder carcinoma and presented with an eczematous lesion involving the penoscrotal region, that was diagnosed as Paget disease in an extramammary site [1]. Subsequently EMPD has been reported involving more frequently the external female genitalia and less commonly, the perianal/perineal region, groin, axilla, umbilicus, eyelids, and also external ear canal [2,3,4].

EMPD has been defined by World Health Organization (WHO) as an intraepithelial neoplasm of epithelial origin expressing apocrine or eccrine glandular-like features and characterized by distinctive large cells with prominent cytoplasm, referred to as Paget cells’ [5].

The pathogenesis of EMPD is not fully understood; the stem cell compartment of the epidermis and hair follicle as well as Toker cells and mammary-like glands have been reported as possible sites of origin of Paget cells [6,7,8].

Over time, different attempts to classify EMPD have been made and, in particular, at the vulvar site, a histopathological classification of VPD has been conceived, distinguishing primary/cutaneous VPD (type 1) from secondary/non-cutaneous VPD [9]. In detail, cutaneous VPD (type 1) is further subdivided according to the presence or absence of dermal invasion: type 1a (intraepithelial disease arising within the epidermis and extending into the epithelium of skin appendages and less commonly arising from the skin appendages and migrate to the overlying epidermis by epidermotropism); type 1b when focal invasion can be observed; type 1c when there is a cutaneous “pagetoid spread” from an underlying vulvar adenocarcinoma of the skin appendage or subcutaneous vulvar glands.

The 5-year survival is highly variable, depending on the entity of infiltration, being, respectively, 100% and 88% for intraepithelial and micro-invasive disease (<1 mm), and only 15% when neoplastic invasion exceeds 1 mm [10].

On the other hand, secondary VPD can originate by epidermotropic metastases or by direct extension from a malignancy of the gastrointestinal tract (type 2) or the uro-genital tract (type 3) [11,12].

More recently, the WHO Classification of Tumours of Female Reproductive Organs (4th edition) considers to use the subdivision of cutaneous and non-cutaneous EMPD in routinary diagnosis [5].

Given the rarity of EMPD, data on genetic alterations are largely unexplored. Findings regarding the hormonal status including Her2/Neu amplification are probably the most studied genetic alteration, likely because of their therapeutic potential but the clinical significance of these abnormalities still remains to be fully understood [13]. Being aware that at present the need of a tailored treatment for EMPD is a critical clinical goal, but its concrete availability is still too far to achieve, we reviewed the current literature in order to study the impact of IHC expression in VPD and EMPD in both genders of biological markers that could serve as potential prognostic/therapeutic factors, including human epidermal growth factor receptor 2 (HER2/neu), Estrogen Receptor (ER), Progesterone Receptor (PR), and Androgen Receptor (AR).

## 2. Materials and Methods

### 2.1. Search Strategy

A systematic literature search (from January 2000 up to June 2020) was performed to identify articles regarding the expression of biological markers in vulvar (VPD) and extra-mammary Paget’s disease (EMPD). Since most published papers before 2000 failed to demonstrate ER, PR, and AR expression, we decided to begin our literature search from 2000, in order to obtain more uniform results. Pubmed, Web of Science, and Scopus were used simultaneously, with the combination of terms (extramammary OR extra mammary OR vulvar) AND (paget OR pagets OR paget’s) AND (molecular OR biological OR marker OR protein OR target OR expression). All articles were initially reviewed by abstract and title browsing to select the relevant reports, which were subjected to further screening.

### 2.2. Study Eligibility

Data retrieved from the studies included the following: author, country, year of publication, sex (% female), total number of cases with vulvar Paget’s disease (VPD) and/or extramammary Paget’s disease (EMPD), mean age, percentage of invasive cases, organ site, and molecular markers expression in immunohistochemistry (IHC). In detail, we selected heterogeneous female and male cases from a series of VPD and EMPD-patients. Our primary aim was to investigate the immunohistochemical expression in both sexes (male and female) of biological markers that could serve as potential prognostic/therapeutic factors, including only human epidermal growth factor receptor 2 (HER2/neu), Estrogen Receptor (ER), Progesterone Receptor (PR), and Androgen Receptor (AR). The language was limited to English.

### 2.3. Data Extraction

Starting from 452 identified references, 109 duplicates were removed. The first step consisted in an accurate reading of titles and abstracts and the analysis of all the references denoted high intra-rate reliability (98.62% agreement; Cohen *K*: 0.97). A total of 54 references were then selected and a full-text assessment was performed. Finally, 27 references which met the eligibility criteria were included in the current work [14,15,16,17,18,19,20,21,22,23,24,25,26,27,28,29,30,31,32,33,34,35,36,37,38,39,40].

**Figure 1:**
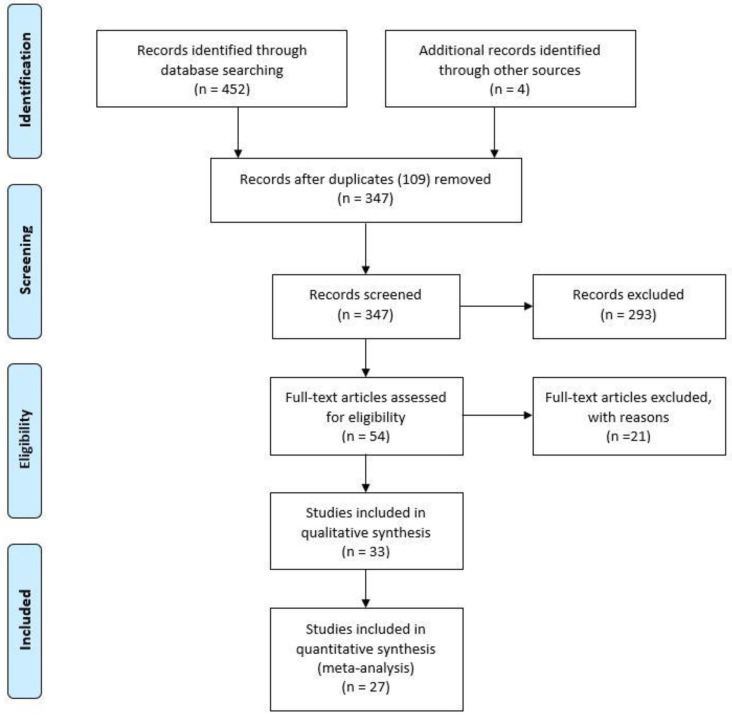
Flow diagram of the study selection process.

The present meta-analysis was conducted according to Guidelines in Preferred Reporting Items for Systematic Reviews and Meta-Analyses (PRISMA) and PICOS (Participants, Intervention, Comparison, Outcomes, Study Design) model. Data from each eligible study were extracted without modification of original data. “Population” of our study was represented by patients diagnosed with VPD/EMPD. “Intervention” (or risk factor) was defined as the VPD/EMPD group with HER2/neu, ER, AR and PR expression, assessed by immunohistochemical analysis. “Comparator” was represented by the VPD/EMPD group without HER2/neu, ER, AR, and PR immunohistochemical expression. Flow diagram of the study selection process is shown in Figure 1.

### 2.4. Risk of Bias across Studies

**Figure 2:**
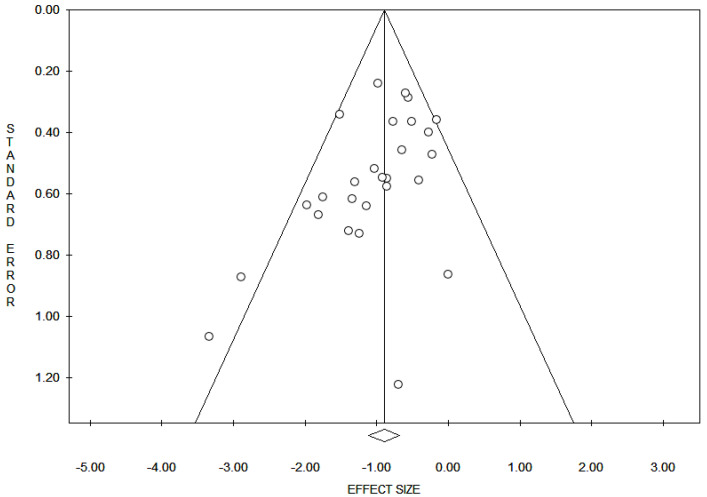
Funnel plot for evaluation of bias across studies. The *x*-axis in the present analysis represents all the markers expression and the *y*-axis represents the standard error. In the absence of bias, a funnel plot should be a symmetrical inverted funnel. In the presence of bias, smaller studies with no expression would be missing, thus creating an asymmetrical funnel. Asymmetry in a funnel plot suggests that there is a systematic difference between larger and smaller studies and/or that there is publication bias.

Reporting bias across studies was evaluated by a graphic diagnostic tool named funnel plot (Figure 2).

### 2.5. Data Analysis

HER2/neu, ER, AR, and PR expression rates across all studies were aggregated using the meta-analytic software ProMeta 2.0 (Internovi, Cesena, Italy). The inverse-variance method was utilized to obtain an overall effect size of the pooled rates of malignancy across studies. Following this, a random effects model was used as a conservative approach to discriminate the different sources of variation among studies (i.e., within-study variance and between-studies variance) [41]. Q and I^2^ statistics were then conducted to evaluate heterogeneity across studies [42]. In detail, a significant Q value denotes the lack of homogeneity among studies; on the other hand, the proportion of observed variance, which indicates real differences in effect sizes was calculated with I^2^ statistics: values of 25%, 50%, and 75% were considered as low, moderate, and high, respectively [43]. Moreover, heterogeneity across study findings was determined using a moderator analysis. Sensitivity analyses were also performed to determine the stability of study results, computing how the overall rates would change by removing one study at a time. Finally, publication bias analyses were established with two tests: the regression method reported by Egger et al. and the Begg and Mazumdar rank correlation test [43,44]. The absence of publication bias is indicated in both tests by non-significant results.

## 3. Results

Based on our criteria, the articles that were published between 2000 and 2020 were analyzed and reported in Table 1. In detail, a total of 27 studies with 713 patients assessed the role of HER2/neu, AR, ER, and PR expression in male and female with VPD and EMPD. The median age was 68 years (range 61–75). The shapes of the funnel plots did not reveal evidence of obvious asymmetry (Figure 1). The results indicated that, in a highly heterogeneous set of 27 studies that compared VPD and EMPD, the overall rate of HER2/neu expression was 30% (95% CI = 0.25–0.36; Q = 34.47; I^2^ = 39.08), the expression’s rate for ER and AR was 13% (95% CI = 0.04–0.36; Q = 17.36; I^2^ = 77.48) and 40% (95% CI = 0.34–0.47; Q = 4.79; I^2^ = 0.00), respectively, and the overall rate for PR was 8% (95% CI = 0.02–0.24; Q = 5.98; I^2^ = 49.79) with *p* < 0.05. The result of publication bias analyses was: Egger test, −1.60; *p* = 0.014; Begg and Mazumdar test, −2.89; *p* = 0.04. Following this, we computed the rate of immuno-markers expression in male and female patients (Table 2). Table 3 illustrates the cut-off values for immunohistochemical markers in the selected studies.

**Human epidermal growth factor Receptor 2 (HER 2/neu)**

The analyses indicated that the expression of HER2/neu in female and male patients was 32% (95% CI = 0.27–0.38) and 26% (95% CI=0.18-0.36), respectively, in a heterogeneous set of 22 studies involving a total of 550 patients.

**Estrogen Receptor (ER)**

The analyses indicated that the expression of ER was 12% (95% CI = 0.03–0.36) in female and 9% (95% CI= 0.00–0.68) in male patients, in a set of five studies involving a total of 118 patients.

**Androgen Receptor (AR)**

The analyses indicated that the expression of AR was 40% (95% CI = 0.34–0.47) in female and 40% (95% CI = 0.32–0.48) in male patients, in a set of seven studies involving a total of 227 patients.

**Progesterone Receptor (PR)**

The analyses indicated that the expression of PR was 9% (95% CI = 0.03–0.25) in female patients in a total set of four studies involving 95 patients. There was only one study that involved five male patients and the rate observed was 2%. Unfortunately, in these cases it was impossible to calculate the heterogeneity’s test.

## 4. Discussion

EMPD, also referred as in situ adenocarcinoma of the skin, is a rare malignant disorder of skin occurring on cutaneous sites with abundant apocrine sweat glands and hair follicles [1,2,3,4,5]. The most common sites of occurrence are represented by the vulvar region, perineal, perianal, scrotal, and penile skin. Axilla, buttocks, thighs, eyelids, and the external auditory canal represent other uncommon sites of occurrence [1,2,3,4,5]. Clinically, EMPD manifests as erythematous or persistent, eczema-like skin lesions [1,2,3,4,5].

The majority of primary EMPD, are confined to the epidermis, with a slow growth and exceptional metastases. However, cases with dermal invasion show an increased propensity for lymph node involvement and distant metastases [45]. In this subset of patients, imaging, ultrasound guided aspirative cytology, as well as sentinel lymph node biopsy have proven interesting results for the early detection of metastases and therapeutic management [46,47,48,49,50].

Before rendering the diagnosis of primary Paget disease, synchronous or metachronous secondary malignancies arising from the underlying dermis and adjacent or distant organs must be taken into consideration. In detail, sweat gland adenocarcinoma, colorectal carcinoma, prostatic carcinoma, endometrioid adenocarcinoma, and urothelial carcinoma represent possible etiologic factors of secondary EMPD [6,9,11,12,51].

In the present review and meta-analysis, we mainly focused on the hormonal environment and HER2 status in EMPD. Surprisingly, all papers before 2000 failed to demonstrate ER, PR, and AR expression, while, starting from 2000, we noted a hormonal background in EMPD mainly dominated by AR (Table 1). In detail, the observed expression rates for ER, PR, and AR were 13%, 9%, and 40%, respectively. Considering the patients’ sex, our results, in a total set of 4 studies involving 95 patients, have shown that the expression of ER was 12% (95% CI = 0.03–0.36) in female and 9% (95% CI= 0.00–0.68) in male patients and that the expression of PR was 9% (95% CI = 0.03–0.25) in female patients, and 2% in male patients. On the other hand, in a set of seven studies involving a total of 227 patients, higher expression rates of AR were detected both in female (40%; 95% CI = 0.34–0.47) and male (40%; 95% CI = 0.32–0.48) patients. According to these findings, anti-androgen target therapy seems promising tool in the management of EMPD [52].

Regarding ER and PR expression in EMPD, limited and conflicting results are still available. However, a recent study by Garganese et al., reported a remarkably high percentage of ER-positive EMPD (at least 70%), which may provide novel insights in the future hormonal treatment of this disease [19].

Regarding HER2 status, our results indicated that, in a highly heterogeneous set of 27 studies, the overall rate of HER2/neu expression was 30% (95% CI = 0.25–0.36; Q = 34.47; I^2^ = 39.08). Considering the patients’ sex, the performed analyses have also indicated that the expression of HER2/neu in female and male patients was 32% (95% CI = 0.27–0.38) and 26% (95% CI=0.18–0.36), respectively. Moreover, some authors highlighted a possible correlation between HER2 overexpression and disease recurrence, dermal invasion, and lymph-node metastases [33,34,35,36].

Few studies have also analyzed HER2 overexpression and gene amplification in metastatic patients. Ogawa et al. have found HER2 overexpression in 19.4% of the lesions, three of which with HER2 amplification by CISH [32]. Tanaka et al. reported that the ERBB2 gene was amplified in all cases with a HER2 score of 3+ [37]. Other authors detected by CISH HER2 gene amplification in 43% of the lesions. HER2 protein overexpression (score 3+ by IHC) was found in 12 tumors (52%), including all 10 tumors with gene amplification [39].

A good overall concordance between HER2 status in primary tumors and in the corresponding metastatic sites has also been described in EMPD [37]. This finding contrasts with the reported discordance rates of HER2 expression between primary and metastatic lesions reported for breast and gastric cancer [53].

According to these results, we can conclude that HER2/neu overexpression is found in at least one-third of EMPD lesions, probably characterized by poor outcome related to deep invasion, recurrence, and node metastases. However, therapies targeting HER2 may be useful in treating HER2 positive advanced and/or metastatic patients [28,36].

Moreover, several studies in the field of epigenetics have documented the pathogenic role of MicroRNAs (miRNAs) in different solid tumors, including HER2 positive breast cancer [54,55,56]. MiRNAs are small endogenous non-coding RNAs with a wide range of cellular functions. In breast cancer, both oncogenic and tumor suppressor properties have been related to specific miRNAs. In detail, miRNAs are involved in different stages of breast cancer progression, such as tumor growth, apoptosis, differentiation, angiogenesis, metastasis, and drug resistance [54,55,56]. Importantly, the tumor suppressor role of miRNAs has been recently highlighted also in HER2-overexpressing breast cancer where they mediate the downstream signaling of HER2, suppress the expression of HER2 and affect responses to anti-HER2 therapies [55].

In this regard, understanding the role of miRNAs in HER2-positive tumors is of great importance for the future development of novel and individualized target-therapies.

## 5. Conclusions

In conclusion, we believe that from the presented meta-analyses some relevant conclusions can be derived: AR status and HER2/neu overexpression/amplification have been shown as two fundamental pathogenetic pathways in both female and male patients affected by EMPD. Moreover, a possible relation between AR/HER2 and tumor invasion/recurrence/metastatic disease have been reported. These findings, anyway, need to be corroborate by further multicentric studies and confirmed by prospective clinical trials using appropriate standardized criteria for hormonal status assessment.

## Figures and Tables

**Table 1:** Characteristics of Included Studies in the Meta-Analysis.

Author	Year	Country	Age (Mean or Median)	Se X(% female)	Total Cases	Micro-Invasive/ Invasive Cases (%)	Positive Expression In Microinvasive/INVASIVE Cases (%)	Marker	Positive ihc Espression (%)	Her2 Amplificatin Status (%)
Aoyagi, et al.	2008	Japan	70.6	34.7	23	6/23 (26)	4/6 (66.6)	HER2	F: 7/8 (87.5) M: 10/15 (66.6)	N/A
Bianco, et al.	2006	USA	75	100	15	N/A	N/A	HER2	6/15 (40)	1/15 (7)
Brummer, et al.	2004	Germany	N/A	100	10	2/10 (20)	2/2 100	HER2	8/10 (80)	N/A
Diaz de Leon, et al.	2000	USA	64.5	82	28	N/A	N/A	AR PR ER	F: 12/23 (52.2) M: 3/5 (60) 0/28 0/28	
Fujimoto, et al.	2000	Japan	67	26.6	30	13/30 (43.3)	9/13 (26.6)	AR	F: 8/8 (100) M: 16/22 (72.7)	
Garganese, et al.	2019	Italy	67	100	41	11/41 (26.8)	HER2 4/11 (36.3) AR 10/11 (90.9) PR 2/11 (18) ER 8/11 (72.7)	HER2 AR PR ER	10/41 (24.4) 33/41 (80.5) 9/41 (22) 29/41 (70.7)	10/41 (24.4)
Gatalica, et al.	2020	USA	61	72.2	18	15/18 (83.3)	AR 9/15(60) ER (4)/15 (26.6)	AR ER	F: 9/13 (69.2) M: 3/5 (60) F: 2/13 (15.3) M: 2/5 (40)	
Hanna, et al.	2003	Canada	N/A	100	20	N/A	N/A	HER2	1/20 (5)	0/19
Hikita, et al.	2012	Japan	70.47	64.70	17	23.5	ER 0/2 PR 0/2 HER2 8/8 4/4(100)	HER2	F: 9/11 (81.8) M: 3/6 (50)	0/8
Horn, et al.	2008	Germany	N/A	100	8	N/A	N/A	HER2 ER PR	8/8 (100) 1/8 (12.5) 1/8 (12.5)	N/A
Inoguchi, et al.	2006	Japan	71.7	17.6	34	N/A	N/A	AR	F: 1/6 (16.6) M: 14/23 (60.8)	
Kasashima, et al.	2010	Japan	71.5	44.8	58	16/58 (27.5)	9/16 (56.2)	AR	F: 12/26 (46) M: 21/32 (65.6)	
Liegl, et al.	2005	Germany	N/A	100	23	N/A	N/A	HER2 AR PR ER	12/23 (52) 18/23 (78) 0/23 (0) 1/23 (4)	N/A
Liu, et al.	2009	USA	69	71.4	14	N/A	N/A	HER2	5/14 (35.7)	N/A
Lu, et al.	2018	China	63	0	11	N/A	N/A	HER2	3/11 (27.2)	2 (FISH+) + 1(genetic heterogeneity)/11
Masuguchi, et al.	2011	Japan	N/A	41.9	31	11/31 (35.4)	10/11 (90.9)	HER-2	F: 7/13 (53.8) M: 12/18 (66.6)	N/A
Miyamoto, et al.	2010	Japan	74	43.7	32	19/32 (59.3)	13/19 (68)	HER2	F: 7/14 (50) M: 13/18 (72)	2/5 (40)
Morbeck, et al.	2016	Brazil	66.8	100	11	2/11 (18)	2/2 (100)	HER2	6/11 (54.5)	2/6 (33.3)
Ogawa, et al.	2005	Japan	68.5	14.7	34	16/34 (47) 18/34 (52.9)	5/18 (27.7)	HER2	F: 1/5 (20) M: 6/29 (20.6)	3/7 (42.8)
Plaza, et al.	2009	USA	66	70.2	47	2/47 (4.2)	0/2 (0)	HER2	F: 14/33 (42.4) M: 1/14 (7)	N/A
Reich, et al.	2005	Austria	63	100	6	N/A	N/A	HER2	4/6 (66.6)	4/6 (66.6)
Richter, et al.	2010	USA	68.5	100	33/39 *	7/33 (21)	5/7 (71)	HER2	19/33 (57.5)	N/A
Sekiguchi, et al.	2020	Japan	71	50	4	N/A	N/A	HER2	F: 2/2 (100) M: 2/2 (100)	2 amplified 2 polysomic
Tanaka, et al.	2016	Japan	72	15.3	26	26/26 (100)	6/26 (23.07)	HER2	F: 2/4 (50%) M: 4/22 (18)	5/6 (83.3)
Tanaka, et al.	2013	Japan	71.1	33.6	104	73/104 (36.5)	10/73 (13.7)	HER2	F: 5/35 (14.2) M: 7/69 (10)	12/16 (75)
Tanskanen, et al.	2003	Finland	65.47	60.8	23	3/23 (13.04)	1/3 (33.3)	HER2	F: 12/23 (52) M: 4/9 (44.44)	10/23 (43.47)
Zhang, et al.	2015	China	61.5	0	2	1/2 (50)	1/2 (50)	HER2	1/2 (50)	N/A

* Tissue specimens available for Her-2/neu testing.

**Table 2:** Summary of meta-analytic results.

	Sex	K	N	Overall Rate of Expression (95% CI), %	Q	I^2^
**Human epidermal growth factor Receptor 2** **(HER 2/neu)**	F M	20 12	341 209	32 (27–38) 26 (18–36)	23.74 26.04	19.98 57.76
**Estrogen Receptor (ER)**	F M	5 2	108 10	12 (3–36) 9 (0–68)	17.30 3.57	76.88 72.02
**Androgen Receptor (AR)**	F M	7 5	140 87	40 (34–47) 40 (32–48)	4.79 0.18	0.00 0.00
**Progesterone Receptor** **(PR)**	F M	4 1	95 5	9 (3–25) 2 (0–22)	5.19 -	42.15 -

**Note.** F: female; M: male; K: number of studies; N: total number of patients; CI: confidence interval; I^2^: index for quantifying the degree of heterogeneity; Q: test for heterogeneity; *p* < 0.001.

**Table 3:** Evaluation of immunohistochemical markers in the selected studies.

Author	Marker	Positive IHC Expression (%)	Evaluation Criteria of IHC
Aoyagi, et al.	HER- 2/neu	F: 7/8 (87.5) M: 10/15 (66.6)	Expression of the antigen was assessed and compared with the reaction in known positive controls semi-quantitatively as follows: <5% of Paget cells positive (score 0); (+) 5–25% of Paget cells positive (score 1); (2+) 26–50% of Paget cells positive (score 2); (3+) >50% of Paget cells positive (score 3).
Bianco, et al.	HER-2/neu	6/15 (40)	Intense staining of tumor cell membranes in the majority of tumor cells was graded as 3+, focal strong membrane staining as 2+, focal low intensity membrane staining as 1+, and granular cytoplasmic staining or no staining of tumor cells as 0.
Brummer, et al.	HER-2/neu	8/10 (80)	In accordance with the Hercep Test kit guide, HER-2/neu overexpression was assessed as negative for scores of 0 and 1+ and positive for scores of 2+ and 3+.
Diaz de Leon, et al.	AR PR ER	F: 12/23 (52.2) M: 3/5 (60) 0/28 0/28	Only nuclear staining with antibodies to steroid receptors was considered specific, and the percentage of cells stained was recorded.
Fujimoto, et al.	AR	F: 8/8 (100) M: 16/22 (72.7)	To roughly measure quantitatively androgen receptor content, a score corresponding to the sum of the percentage of tumor cells stained (0, no staining; 1, less than 25%; 2, 26–50%; 3, 51–75%; 4, 76–100%) and the staining intensity (0, absent; 1, weak; 2, moderate; 3, strong) was established
Garganese, et al.	HER-2/neu AR PR ER	10/41 (24.4) 33/41 (80.5) 9/41 (22) 29/41 (70.7)	For HER2/neu expression, membrane staining was evaluated according to the ASCO-CAP guidelines described for breast cancer. A tumor showing ER, PR, or AR nuclear staining in a fraction of neoplastic cells ≥ 1% was considered positive.
Gatalica, et al.	AR ER	F: 9/13 (69.2) M: 3/5 (60) F: 2/13 (15.3) M: 2/5 (40)	AR, ER were analyzed using a ≥ 10% threshold for nuclear positivity.
Hanna, et al.	HER-2/neu	1/20 (5)	Results for HER-2/neu status by immunohistochemistry were reported as follows: positive when at least 10% of the tumor showed moderate/strong complete membrane staining, negative when less than 10% of the tumor showed complete membrane staining or less than 30% showed weak or incomplete membrane staining, and equivocal when ≥ 30% of cells showed diffuse weak staining
Hikita, et al.	HER-2/neu	F: 9/11 (81.8) M: 3/6 (50)	For HER2/neu expression, membrane staining was evaluated according to the ASCO-CAP guidelines
Horn, et al.	HER-2/neu ER PR	8/8 (100) 1/8 (12.5) 1/8 (12.5)	According to the recommendation for breast cancer, a tumor was counted as positive if a minimum of 10% of the cells showed positive intranuclear staining regardless of staining intensity. For HER-2/neu, only membranous staining results were scored using the system recommended in breast cancer
Inoguchi, et al.	AR	F: 1/6 (16.6) M: 14/23 (60.8)	The stained sections were evaluated microscopically as follows: − = no staining; + = focal deposition in the nest of tumor cells.
Kasashima, et al.	AR	F: 12/26 (46) M: 21/32 (65.6)	Immunopositive labelling of ≥10% among all cells was considered as a positive result
Liegl, et al.	HER-2 AR PR ER	12/23 (52) 18/23 (78) 0/23 1/23 (4)	For HER2/neu expression, membrane staining was evaluated according to the ASCO-CAP guidelines described for breast cancer. A tumor showing ER, PR, or AR nuclear staining in a fraction of neoplastic cells ≥ 1% was considered positive.
Liu, et al. 2018	HER-2/neu	5/14 (35.7)	For HER2/neu expression, membrane staining was evaluated according to the ASCO-CAP guidelines
Lu, et al.	HER-2/neu	3/11 (27.2)	For HER2/neu expression, membrane staining was evaluated according to the ASCO-CAP guidelines
Masuguchi, et al.	HER-2/neu	F: 7/13 (53.8) M: 4/18 (22.2)	Grading system: 1+ for slight staining, 3+ for strong staining, and 2+ for staining between 1+ and 3+.
Miyamoto, et al.	HER-2/neu	F: 7/14 (50) M: 13/18 (72)	Only membrane staining was evaluated using the 0 to 3+ scale illustrated in the HercepTest scoring guideline (0 for no staining or membrane staining in less than 30% of the cells; 1+ for partial, weak staining of the cell membrane in 30% of the cells; 2+ for moderate staining of the complete cell membrane in 30% of the cells; 3+ for intense staining of the complete membrane in.30% of the cells
Morbeck, et al.	HER-2/neu	6/11 (54.5)	For HER2/neu expression, membrane staining was evaluated according to the ASCO-CAP guidelines
Ogawa, et al.	HER-2/neu	F: 1/5 (20) M: 6/29 (20.6)	3+: more than 10% of tumor cells show strong complete membrane staining; 2+: more than 10% of tumor cells show weak or moderate and complete membrane staining; 1+: positively stained cells are less than 10% or show only faint staining, although more than 10% of cells are positive; 0, no staining. According to the recommendation of the US Food and Drug Administration, 3+ and 2+ were recorded as overexpressed and 1+ and 0 as non-overexpressed.
Plaza, et al.	HER-2/neu	F: 14/33 (42.4) M: 1/14 (7)	no staining or membrane staining in less than 10% of the cells; 1+ for partial, weak staining of the cell membrane in. 10% of the cells; 2+ for moderate staining of the complete cell membrane in 10% of the cells; 3+ for intense staining of the complete membrane in 10% of the cells). Overexpression was assessed as positive for scores 2 and 3+ and negative for scores 0 and 1+.
Reich, et al.	HER-2/neu	4/6 (100)	For HER2/neu expression, membrane staining was evaluated according to the ASCO-CAP guidelines
Richter, et al.	HER-2/neu	19/33 (57.5)	Staining was considered to be 0 for no staining, 1+ with less than 10% positively stained cells or cells that showed only faint staining, although more than 10% of cells were positive, 2+ when they showed more than 10% weak or moderate and complete membrane staining, and 3+ when more than 10% of tumor cells showed strong complete membrane staining.21 According to the standard guideline used for breast cancer, 2+ and 3+ tumors were recorded as ‘overexpressed’, 0 and 1+ tumors as ‘non-overexpressed’
Sekiguchi, et al.	HER-2/neu	F: 2/2 (100) M: 2/2 (100)	For HER2/neu expression, membrane staining was evaluated according to the ASCO-CAP guidelines
Tanaka, et al.	HER-2/neu	F: 2/4 (50) M: 4/22 (18)	3+, strong, complete membrane staining in more than 30% of the malignant cells; 2+, weak to moderate complete membrane staining in more than 10% of the malignant cells or strong, complete membrane staining in more than 10–30% of the malignant cells; 1+, weak to moderate incomplete membrane staining; 0, fewer than 10% of membrane staining or no membrane staining. Those cases scored 0 and 1+ were defined as negative. Cases scored 2+ and 3+ were defined as equivocal and overexpression, respectively
Tanaka, et al.	HER-2/neu	F: 5/35 (14.2) M: 7/69 (10)	3+, strong, complete membrane staining in more than 30% of the malignant cells; 2+, weak to moderate complete membrane staining in more than 10% of the malignant cells or strong, complete membrane staining in more than 10–30% of the malignant cells; 1+, weak to moderate incomplete membrane staining; 0, fewer than 10% of membrane staining or no membrane staining. Those cases scored 0 and 1+ were defined as negative. Cases scored 2+ and 3+ were defined as equivocal and overexpression, respectively
Tanskanen, et al.	HER-2/neu	F: 12/23 (52) M: 4/9 (44.44)	For HER2/neu expression, membrane staining was evaluated according to the ASCO-CAP guidelines
Zhang, et al.	HER-2/neu	1/2 (50)	For HER2/neu expression, membrane staining was evaluated according to the ASCO-CAP guidelines
